# Reactive Arthritis After COVID-19: A Case Report

**DOI:** 10.7759/cureus.24096

**Published:** 2022-04-13

**Authors:** Mohammed Basheikh

**Affiliations:** 1 Department of Medicine, Faculty of Medicine, King Abdulaziz University, Jeddah, SAU

**Keywords:** arthritis, conjunctivitis, balanitis, covid-19, reactive arthritis

## Abstract

A 43-year-old healthy male was diagnosed with symptomatic COVID-19. Soon after recovery, he experienced severe back pain, bilateral red eye, and a new penile lesion. He was diagnosed with reactive arthritis, given he presented with arthritis, conjunctivitis, and balanitis.

The patient was treated with nonsteroidal anti-inflammatory drugs (NSAIDs), a short course of systemic steroids, and a local steroid cream on the penile lesion, followed by a local antifungal cream for two months. The patient responded well to the treatment and returned to his usual life activities.

## Introduction

COVID-19 has caused a substantial burden to healthcare systems worldwide. The disease has affected the lifestyles of people, which might take a long time to recover. The disease presents with many symptoms, including fever, upper and lower respiratory symptoms, gastroenterological symptoms, and general malaise, among others [1].

The typical immune response to the disease can lead to several complications, including thrombotic events, chronic lung disease, prolonged intensive care unit and hospital stay, and mortality [2]. Some emerging and experimental treatment modalities also have consequences with as yet unknown side effects [1]. Furthermore, some known syndromes have become associated with COVID-19, such as reactive arthritis [3,4]. Here, we describe the case of a male patient who developed reactive arthritis after recovery from SARS-CoV-2 infection.

## Case presentation

In July 2021, a 43-year-old Arab male developed a sore throat two days after interacting with his father, who was diagnosed with SARS-CoV-2 infection. A throat swab test was done, which tested positive. Later that day, he started to develop a high-grade fever of 39°C. Given that he had no respiratory symptoms and was considered a mild COVID-19 case, he was sent home for isolation with supportive care. At home, he started to have rhinorrhea, congested nose, and myalgia. He was started on regular paracetamol 1 g every six hours, but the myalgia persisted. He started self-administering ibuprofen 400 mg twice a day as he started to become bedbound and unable to walk. On day 4 after the onset of symptoms, he developed anosmia and ageusia. After two days of taking ibuprofen, his mobility and symptoms improved, but the anosmia and ageusia remained. On day 7 after the onset of COVID-19 symptoms, he stopped all medications and continued to do fine. On day 10, a throat swab was repeated on patient preference, and the result came back negative. On day 15, he started to develop red eyes, which he started treating with artificial tears. On day 16, he started to complain of severe lower back pain and noticed the appearance of a painless penile lesion with dysuria, which led him to seek medical advice.

**Figure 1:**
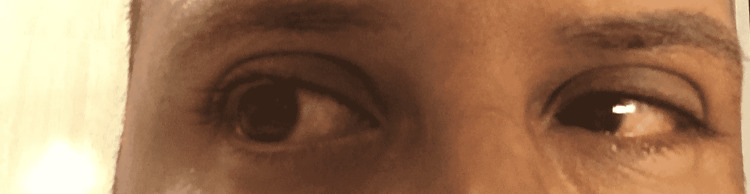
Bilateral conjunctivitis presentation in the patient after recovery from COVID-19.

**Figure 2:**
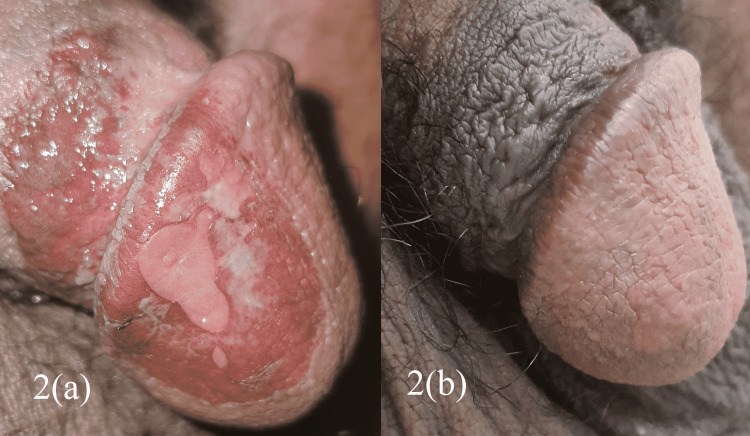
Circinate balanitis at (a) presentation and (b) two months after treatment.

The patient had no known chronic medical diseases and was not on regular medications. Furthermore, he had no family history of autoimmune disease. He is married and has extramarital relationships. His back pain started suddenly on day 15; the pain is worse at rest, especially at sleeping time. The patient was bedbound due to the pain. He feels the pain in the lower back and buttock area. On examination, his vital signs were within normal range, and he was classified as obese class I with a body mass index of 34. He was formally diagnosed with bilateral conjunctivitis (Figure 1), focal tenderness in the sacroiliac area, and circinate balanitis (Figure 2). 

Further examination showed normal complete blood count, electrolyte levels, renal function, and liver and thyroid functions. Erythrocyte sedimentation rate was 40 mm/hour, which was mildly elevated, and C-reactive protein was also elevated at 8 mg/L. Antinuclear antibodies and rheumatoid factor were both negative. Screening for sexually transmitted diseases was negative. Urine analysis and culture were negative for bacteria, and sacroiliac joint X-ray was unremarkable.

The patient was started on treatment for reactive arthritis with ibuprofen as a nonsteroidal anti-inflammatory agent (starting dose: 600 mg every eight hours) and prednisolone as a systemic steroid (25 mg once daily for five days), with a local steroid for the penile lesion. The combination of ibuprofen and steroids was started because of the severity of the symptoms, given all reactive arthritis symptoms are present and the fact that the patient's functional status was poor because of his symptoms. Two days after the start of treatment, the lower back pain and dysuria improved significantly. At one-week follow-up, dysuria had completely disappeared, but a whitish discharge developed on the penile lesion, likely a local fungal infection. The patient was advised to adhere to personal hygiene and use regular soap for pelvic area cleaning. He was also started on antifungal terbinafine 1% cream applied locally twice a day. At one-month follow-up, significant improvement was observed in the penile lesion, and the patient was further advised to continue the treatment for six weeks. The patient reported no more symptoms at follow-up after two months, and his penile lesion disappeared (Figure 2). HLA-B27 test was done in follow-up, and the result was negative. Consent was obtained from the patient prior to the writing of the manuscript.

## Discussion

Reactive arthritis is a syndrome of post-genitourinary or gastroenterological infections. It mainly presents as the triad of arthritis (peripheral or axial), conjunctivitis, and genitourinary symptoms, especially circinate balanitis [5]. Usually, it is more common with genitourinary and digestive bacterial infections such as chlamydia, shigella, and campylobacter.

Since the start of the COVID-19 pandemic in late 2019, few cases of reactive arthritis related to COVID-19 have been reported, most of which were in men aged 40-70 years [6]. COVID-19 has been found to trigger a strong immune response in the body, which could explain the development of reactive arthritis. In one case, the patient presented four days after being diagnosed with COVID-19 with severe pain in multiple joints and lower limbs [4]. In another case, the patient presented with right knee pain and penile lesion and then was diagnosed with COVID-19 [6].

In the present case, the patient experienced the triad of reactive arthritis shortly after recovering from COVID-19 infection. Additionally, the patient presented with symptoms that were not described in the other case report for reactive arthritis after COVID-19 infection [3,4,6], in particular conjunctivitis and balanitis. Possible sexually transmitted infections were ruled out. The response to the treatment was quick and evident with the use of nonsteroidal anti-inflammatory drugs (NSAIDs) and a short course of glucocorticoid.

The first line of treatment is NSAIDs [7]; however, a glucocorticoid administration is initiated when the response is poor [7]. If the patient develops chronic reactive arthritis, a disease-modifying agent could be considered after consultation with a rheumatologist [7].

Given the recent start of the COVID-19 pandemic compared to other outbreaks and pandemics, still, a lot of outcomes are not known to medical teams. Several patients experience fatigue symptoms and mobility complaints [4,6]. It will take several years to know the full extent of the COVID-19 pandemic outcomes.

## Conclusions

The patient described in the present report had reactive arthritis after COVID-19 infection, which presented with a triad of symptoms. However, the patient responded exceptionally to the combination of systemic steroids and NSAIDs over a short treatment course of five days. It is our understanding that the early recognition and treatment of reactive arthritis ensured a favorable outcome. Additionally, circinate balanitis requires regular follow-up examinations as complications such as fungal infections may arise. Furthermore, personal hygiene and the application of local antifungals are imperative in addressing this disease.

Reactive arthritis can severely limit movement and affect the quality of life. Medical teams need to address and manage such cases as soon as they are recognized, especially if they develop after COVID-19 infection, as this will have a huge impact on the quality of life of patients with reactive arthritis.

It is unclear whether the COVID-19 vaccine can cause this side effect; furthermore, it is unclear whether COVID-19 has any possible effect on the occurrence of reactive arthritis with or without vaccine administration. Further studies on the association between SARS-CoV-2 and reactive arthritis are warranted.
